# Whole-genome demography of COVID-19 virus during its pandemic period and on “panvalent” vaccine design

**DOI:** 10.1038/s41598-024-68432-5

**Published:** 2024-08-01

**Authors:** Byung-Ju Kim, JaeJin Choi, Sung-Hou Kim

**Affiliations:** 1 Department of Chemistry and Center for Computational Biology, University of California, Berkeley, CA 94720 USA; 2 Convergence Research Center for Insect Vectors, Incheon National University, Incheon, 22012 Republic of Korea; 3 Graduate Program of Comparative Biochemistry, University of California, Berkeley, CA 94720 USA; 4 Division of Biological Systems and Engineering, Lawrence Berkeley National Laboratory, Berkeley, CA 94720 USA; 5 Department of Molecular Biophysics and Integrated Bioimaging Division, Lawrence Berkeley National Laboratory, Berkeley, CA 94720 USA; 6 Present Address: Department of Dermatology, Chobanian and Avedisian School of Medicine, Boston University, Boston, MA USA

**Keywords:** Vaccines, Classification and taxonomy, Computational biology and bioinformatics, Immunology

## Abstract

With over 16 million submitted genomic sequences, the SARS-CoV-2 (SC2) virus, the cause of the most recent worldwide COVID-19 pandemic, has become the most sequenced genome of all known viruses, revealing, for example, a vast number of expanding viral lineages. Since the pandemic phase appears to be over, we performed a retrospective re-examination of the demographic grouping pattern and their genomic characteristics during the *entire* pandemic period up to the peak of the last pandemic wave. For our study, we extracted from the NCBI *only unique viral sequences* and converted each sequence data to a *relational* vector, indicating the presence/absence of each *variational event* compared to a “reference” sequence. Our study revealed several genomic features that are unexpected or different from those of previous studies. For example, approximately 44,000 variants with unique sequences emerged during the pandemic period; they group into only four major viral-genomic groups and each has a set of mostly unique highly-conserved variant-genotypes (HCVGs); and a small set from the first (“ancestral”) group was inherited by the three (“descendant”) groups, suggesting that HCVGs in the next group may be *predictable* from the current group(s). Such a concept may be potentially important in designing “panvalent” vaccines against the current and future waves of viral infections.

## Introduction

SARS-CoV-2 (SC2) is a positive-sense single-stranded RNA virus that encodes approximately 27 proteins. It was identified as the cause of the most recent worldwide pandemic, COVID-19, which started in December 2019 and is currently downgraded to an endemic virus. Extensive and intensive studies on various aspects of the virus and its short and long-term effects on human health are ongoing. To date, tens of thousands of variants of SC2 with unique sequences, each appearing many times, have been identified^1,2^. Since the pandemic period seems to be over, we can retrospectively re-examine all pandemic variants *together* for their degree of sequence diversity, global demographic grouping, differences in characteristics among the groups and others which may provide observational bases for understanding the various aspects of SC2 during its pandemic period and for predicting and preventing its possible future resurgence.

For our study we started with approximately 380,000 complete sequences longer than the full coding region with no ambiguous nucleotide-calling (see Table S1 in B. Supplementary Figures and Tables of Supplementary Information (SI)), available from the National Center for Biotechnology Information (NCBI; 2), covering up to the time of the peak of the last pandemic wave in February 2022. We aligned them to find the “extended coding region (ECR)” among them, which includes the complete coding region of the virus and parts of untranslated regions beyond the 5’ and 3’ ends of the coding region (see Fig. S1 in B. Supplementary Figures and Tables of the SI). We then assembled two types of input data sets: (a) a curated *unique sequence* data (approximately 44,000) of the ECRs, each of which represents the first sample among those with the same ECR sequence (FES) (i.e., the earliest-collected sample among the subgroup members with the same unique sequence) of the ECR (see Table S1 in B. Supplementary Figures and Tables of the SI) and (b) a relational binary data where we convert the sequence of the ECR of each virus in (a) above into a “binary bit-vector” representing the presence/absence of a *variational event* at each position as compared to that of the reference sequence. The relational data were used to dramatically reduce the massive computational load of a large amount of data as well as to directly relate sequence variations to variational events (see Binary bit-vector representation for genotype variations in A. Detail Materials and Methods of the SI) during the search for global clustering patterns of approximately 44,000 unique FES sequences (44K data). Such relational-vector representation has not been applied in other similar studies of SC2^3–9^.

### Observations

The various genomic features described in this study are based on the data obtained during the period between the emergence of the first wave and the peak of the last pandemic wave, when the SC2 pandemic started to subside. Our observations and analyses are described at several different levels of genomic demography during this period. Since some features, observed at the whole population or major genomic group (MGG) level, are caused by the features observed at one or more deeper levels of individual MGG or individual virus level, we numbered all 13 notable genomic features contiguously.

**Figure 1:**
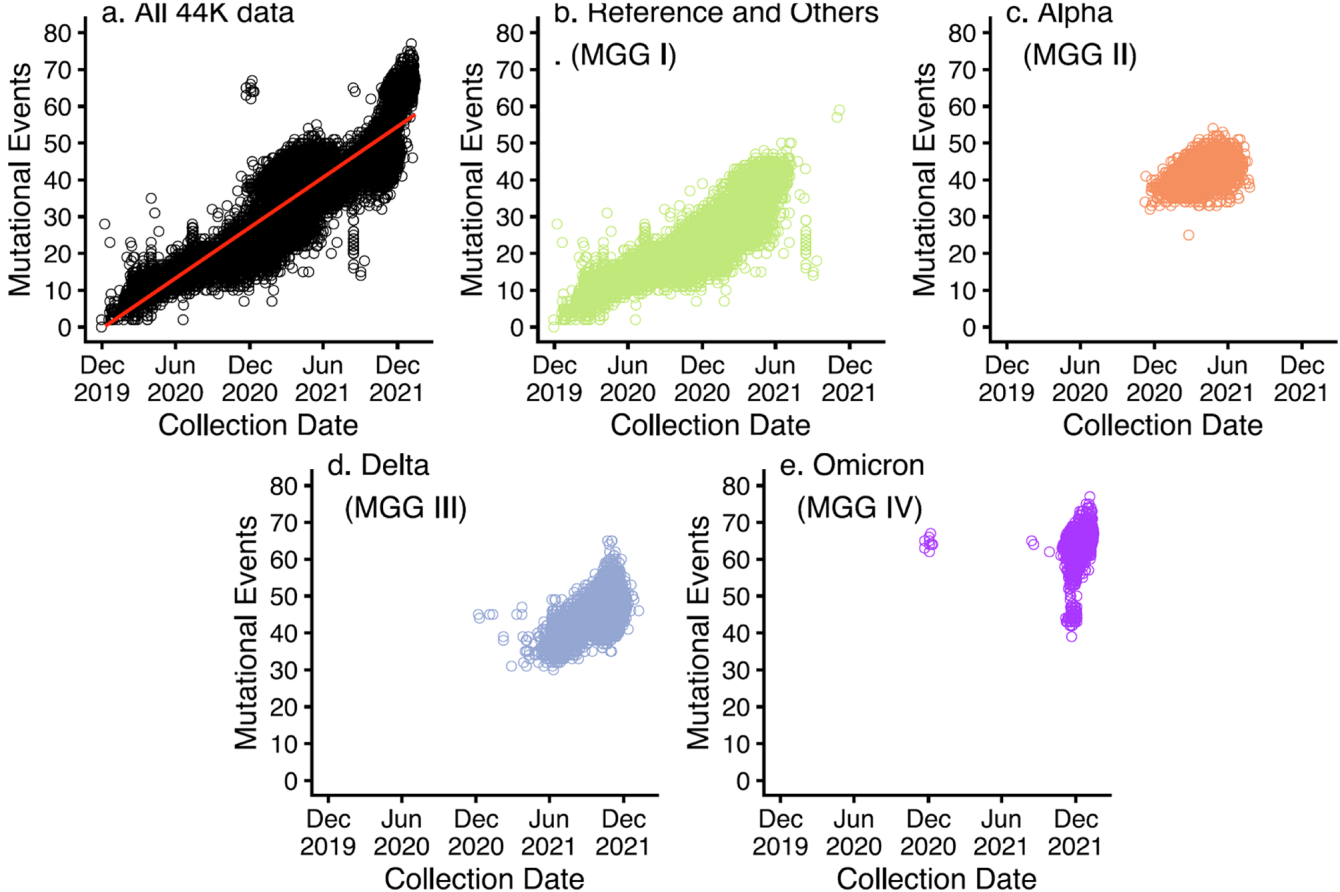
Number of unique variational events vs. emergence time of new variants (sample-collection date of the first sample of a subpopulation with a unique sequence). (**a**) All 44K samples, (**b**) MGG I population, (**c**) MGG II (mostly alpha variants) population, (**d**) MGG III (mostly delta variants) population, and (**e**) MGG IV (mostly omicron variants) population.

**At the level of the whole population**:

1. There are approximately 44,000 unique sequence-variants of SC2. An examination of the curated whole-genome sequences of 406,004 SC2 viruses (from the NCBI database^2^ as of the peak of the last pandemic wave around February 2022) revealed that most of them are highly redundant for the ECR. We identified 43,678 *unique* sequences (called “44 K” data) that we call “reliable”, based on our filtering criteria (see Table S1 in B. Supplementary Figures and Tables of the SI). Thus, approximately 89% of the whole sequences are the duplicate sequences of 11%, which have unique sequences different from the reference sequence of the Wuhan-Hu-1 strain^2^, which was chosen as the reference sequence in this study.
2. Mutational variations are found at 63% of the ECR positions of the reference sequence. Mutational events occurred very broadly, covering 18,655 positions of the ECR of the reference, which is approximately 63% of 29,549 ECR sequence positions (see Table S2 in B. Supplementary Figures and Tables of the SI). An overwhelming portion of the mutational positions, most of which are substitutional mutations, showed very low (10% or lower) mutational frequencies in each MGG. However, there are 104 positions (approximately 0.35% of the ECR) where the frequencies of the unique mutated-genotypes are extremely high in each MGG (approximately 90% or higher; see Observation #10 below).
3. There are only two types of variational events. In the 44 K data, there were only two types of variational (mutational) events observed during the pandemic period: the overwhelming portion (99.6%) of all variational events were point substitutions, and the remaining variational events were short indels, approximately 90% of which were deletional events (see Table S2 in B. Supplementary Figures and Tables of the SI).
4. One to 77 variational events occurred per virus. Figure 1a shows that, despite the vast number of variants, the number of genomic variational events per unique ECR ranged from one to 77 by the time of the peak of the last pandemic wave (February 2022). This rate corresponds to the average (red line) accumulation of 29 variational (mutational) events per virus per year or approximately one variational event per 1000 loci per year.

**At the level of four major genomic groups (MGGs):**

**Figure 2:**
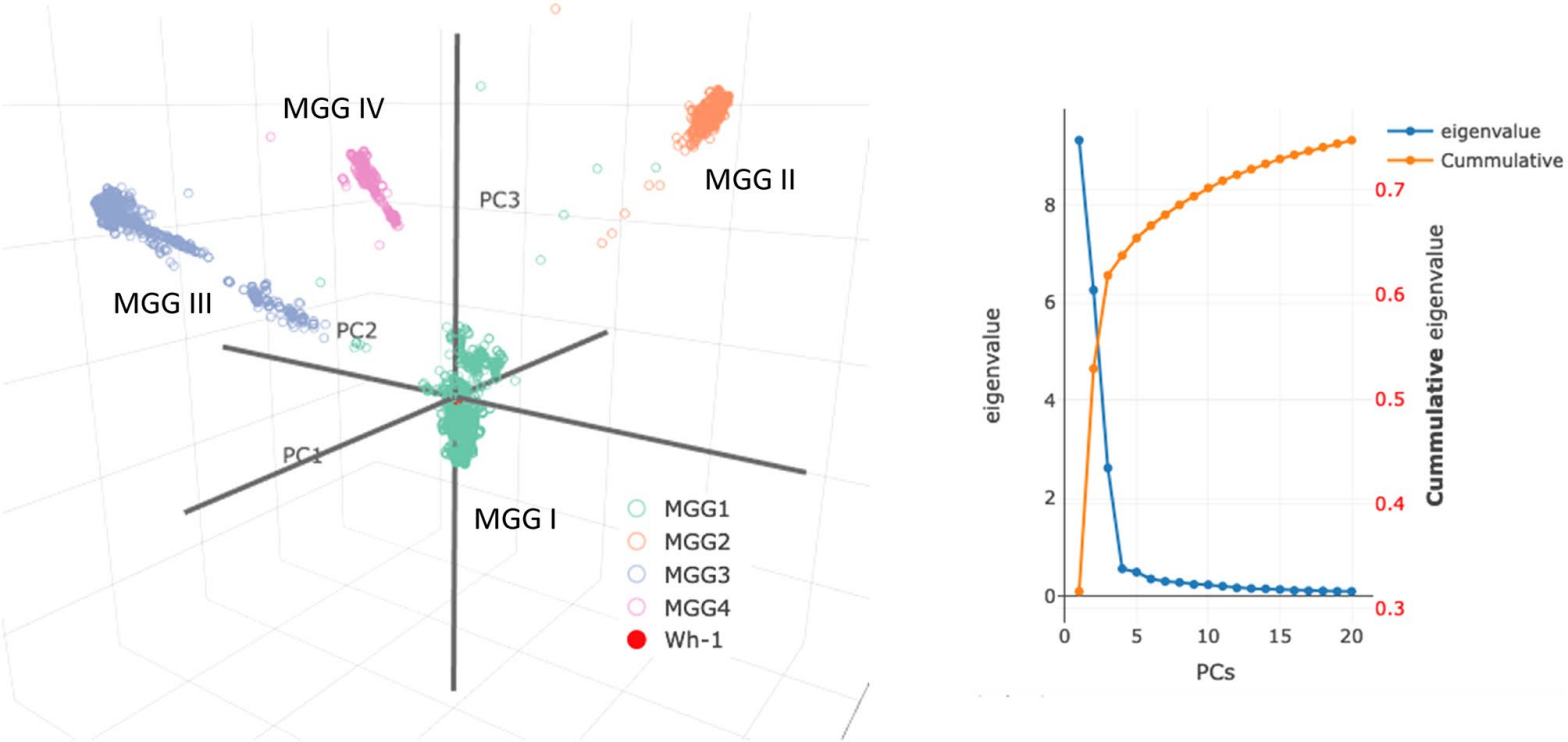
(Left). (**A**) plot of principal component analysis (PCA) of the 44K variants with unique sequences reveals four well-segregated major clusters, named Major Genomic Group (MGG) I, II, III, and IV (also see Figure S2 (video) in (**B**) Supplementary Figures and Tables in Supplementary Information (SI). When colored by the Variants of Concerns (VOCs) of World Health Organization (WHO)^7^, it reveals that MGGs II, III and IV consist of almost all alpha, delta, and omicron variants, respectively, and MGG I consists of all the remaining variants (collectively shown in green, for convenience) and the reference group (shown in red within green near the origin). (Right). Normalized eigenvalues are shown in black on the left Y-axis and the cumulative normalized eigenvalues are shown in red on the right Y-axis. In this plot, the input format of each variant is represented by a binary bit-vector (see Binary Bit-vector Representation of Sequence in Materials and Methods). For a video, see Fig. S2 in B. Supplementary Figures and Tables of the SI.

**Figure 3:**
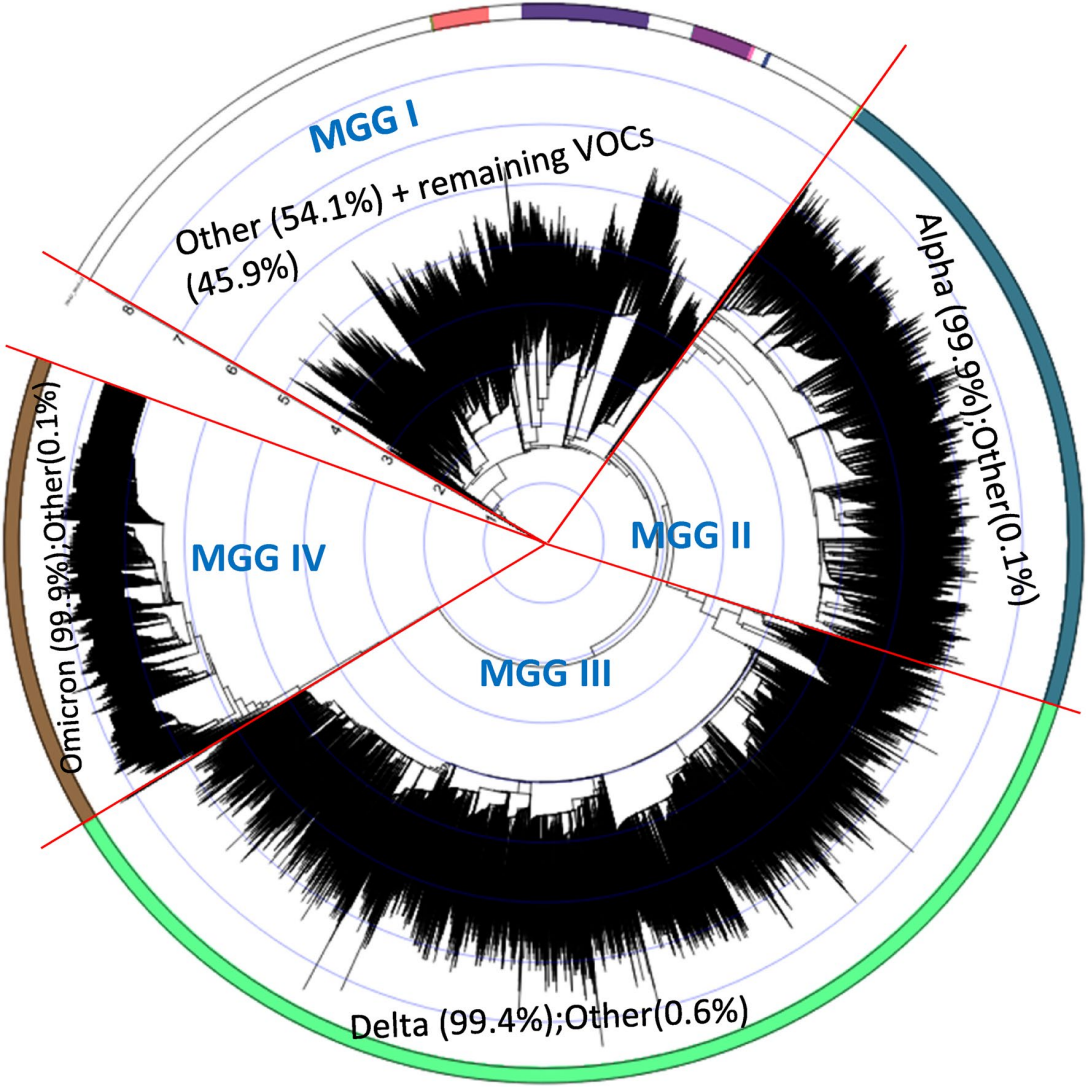
A circular representation of a BIONJ phylogenetic tree^11^ of the 44K variants with unique sequences reveals four major clades corresponding to MGG I–IV in the PCA plot shown in Fig. 2. In this tree, the input format is a square matrix of Euclidean distances of pairwise binary bit-vectors (see Binary Bit-vector Representation of Sequence in Materials and Methods).

5. Four major genomic “clusters” based exclusively on genomic variational distance. Figure 2 shows the plot of principal component analysis (PCA) (^10^; see Principal Component Analysis (PCA) in A. Details of Materials and Methods in the SI) on the entire 44 K data set. By the time of the peak of the last pandemic wave, *all* the unique genomic variants in the study could be grouped into four major clusters that were well segregated in the PCA space, exclusively based on genomic variational distances (defined as Euclidian distances between two binary bit-vectors) *without* assuming any evolutionary model. We named them Major Genomic Group (MGG) I, II, III, and IV (also see Fig. S2 (video) in B. Supplementary Figures and Tables of the SI). Among the many different labels we tested, the Variants of Concerns (VOCs) of the World Health Organization (WHO)^7^ showed the simplest correlation: MGG II, III and IV consisted of almost exclusively Alpha, Delta, and Omicron variants of WHO, respectively, and MGG I consist of a mixture of the rest (other earlier VOCs and those not belonging to any designated VOCs including the reference group). When each MGG is magnified (not shown) it consists of multiple subgroups, each of which appears to be related to a different lineage, where members are separated by one or a few variational events in PCA space.
6. Four major genomic “clades” based on a “phylogenetic” tree of the genomic variational distance *and* evolutionary model. Using the clading portion of the simple tree building on the entire 44 K data set, Fig. 3 shows a circular representation of a phylogenetic tree built using the Euclidian distances between the binary bit-vectors under the *assumptions of a particular evolutionary model* of bifurcating/neighbor-joining and maximum parsimony of branch-lengths (BioNJ^11^; see the BioNJ tree in A. Details of Materials and Methods of the SI). The figure also reveals four major “clades” of the tree, corresponding approximately to the four MGGs of the PCA in Observation #5 above.
7. Pandemic waves (except the first wave) consisted of the variants from more than one MGG. The pandemic came in several waves. As shown in Fig. 1a, although the *average number* of unique sequences of the emerging variants gradually increased as the pandemic progressed (the red line in Fig. 1a), the ranges of the number of the variants undulate like waves as the pandemic progressed. Furthermore, since all the subsequent variants of the same sequence (not represented in Fig. 1) continue propagating as the pandemic progresses, each wave (except the first wave), at most time points, consists of the variants from more than one MGG as shown in Table S3 in B. Supplementary Figures and Tables in the SI. This observation becomes relevant in the Implications and Discussion: “Panvalent” vaccine design against the current and next wave(s) below.
8. “Co-emergence” of *the founders* of MGG II, III, and IV: Although the *centers* of the four MGG populations appear to have emerged sequentially (see the red line in Fig. 1), there are three lines of evidence for the approximate “co-emergence” of the *founders* of the last three MGGs:
  - Based on sample collection-dates: Each panel in Figure 1a–e shows the sample-collection date of each FES (the first sample of each subgroup with a unique ECR sequence) on the X-axis vs. the count of genomic variational events on the Y-axis. These findings suggest that the *members* of MGG I evolved during a period of one and a half years (see Fig. 1b). Then, those of MGG II and MGG III (see Fig. 1c,d), as well as the “early” MGG IV (a small isolated subcluster located far left of the major population cluster of the MGG IV, the “late” MGG IV (see Fig. 1e)), emerged at *almost the same time* around Dec. 2020.
  - Based on the cumulative branch lengths in the phylogenetic tree: An examination of the cumulative branch lengths of the founders of the three MGGs (II, III, and IV) in Fig. 3 shows that they are very similar, suggesting that the three founders emerged within a short time range.
  - Most of the loci of very highly conserved variant-genotypes (evolutionarily selected) of each of the 3 late-emerging MGGs were not correlated with each other (see Observation #10 below) suggesting that each of the three MGGs evolved independently.

**At the level of the individual Major Genomic Group:**

**Figure 4:**
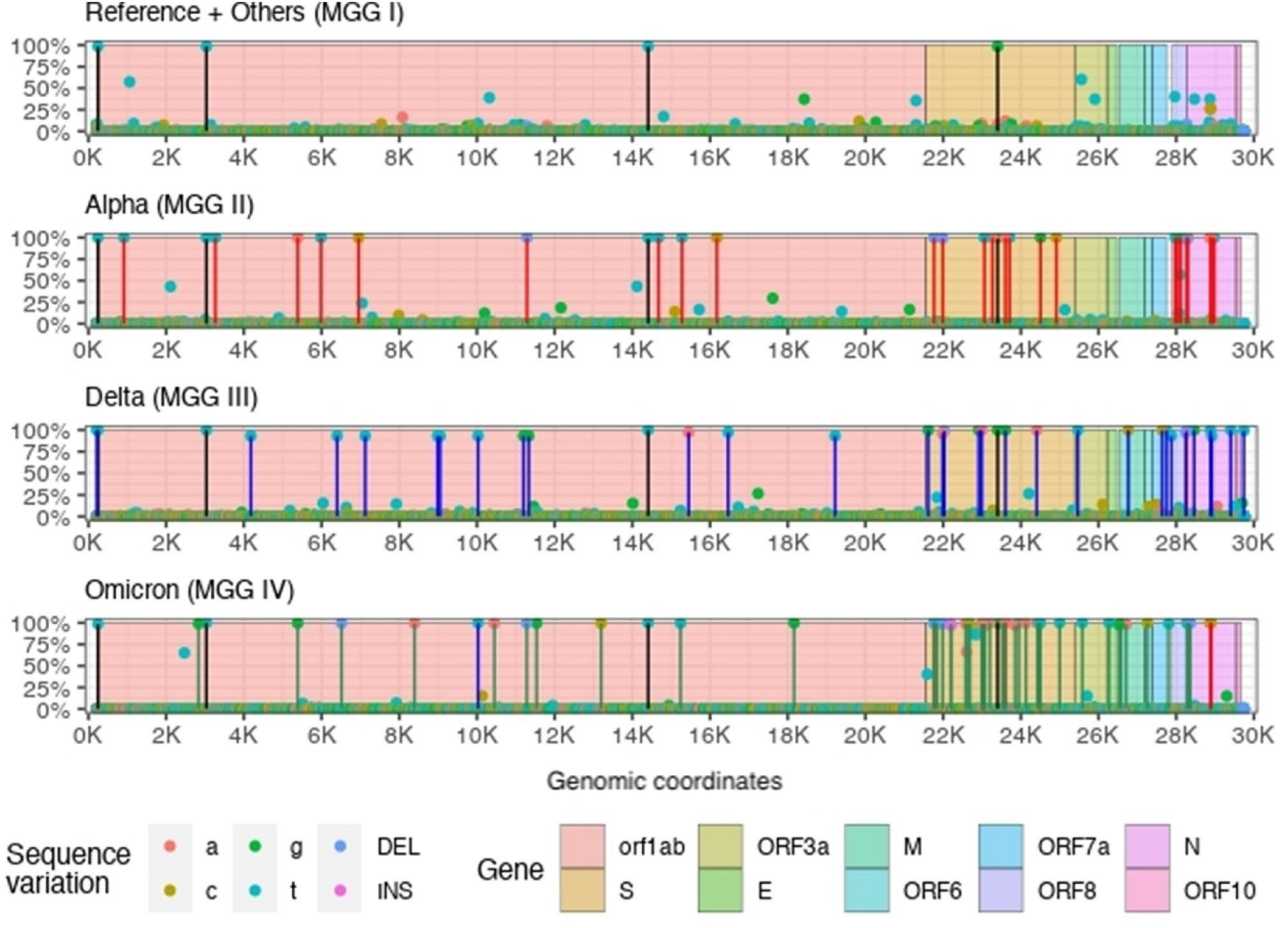
Percent frequencies of unique variant genotype at each locus (on the X-axis) in each MGG. For computational efficiency, 1000 nonoverlapping samples were randomly selected multiple times (7, 8, 16 and 6 times for MGG I, II, III and IV, respectively), and the average % frequency for each locus (small sphere) is shown on the Y-axis. The vertical thick lines with small spheres on top show the loci where the frequency is greater than approximately 90%. For faster and simplified plotting, mutation frequencies < 1% are not shown.

**Figure 5:**
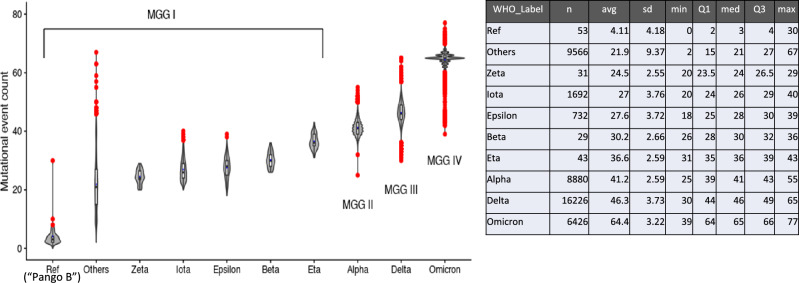
A plot combining a box plot and a violin plot of the distribution of mutational event count for the four MGGs. For each subpopulation, the blue-filled circle near the middle of each rectangle represents the average mutational event, and the red dots are “outliers” based on quartile statistics (see the inset on the right). On the X-axis, MGG I is subdivided into several minor WHO labels; “Others” are for the samples not labeled by the WHO; and the “Ref” subgroup corresponds to the Pango lineage B, which contains most of the variants from Wuhan, China. The Y-axis indicates the number of variational events each sample acquired.

9. Bimodal distribution of mutational frequencies: The loci of mutations in all four MGGs are dispersed throughout the ECR with no immediately obvious clustering (see Fig. 4). However, at a given locus of each MGG, the distribution of the *frequency* of each variational event is highly bimodal: the frequency of occurrence of any variation at most of all loci are lower than 10% (rarely conserved, i.e., random events as indicated by a green “band” formed by contiguous green dots) of the members of each MGG population, but for a very few loci, the frequency is more than 90%, i.e., very highly conserved or selected (see Observation #10 below), as shown by the vertical lines with dots on top.
10. Highly-conserved variant-genotypes (HCVGs) for each MGG: Examination of the genotypes of each MGG in detail revealed that each MGG has a *very small number* of selected loci with HCVGs, which are present only among all variant viruses, but not among the founding viruses. These HCVGs have the following interesting characteristics and implications (also see Implications and Discussion below):
  - 129 HCVGs: Fig. 4 shows that, at 4, 32, 35 and 58 loci of MGG I, II, III, and IV, respectively, each locus has an HCVG (approximately 90% or more of its members of each of the four MGGs, shown as vertical lines with dots on top, have conserved variant genotypes). These 129 HCVGs among the four MGGs are found at 104 unique loci (i.e., the union of 129 loci from 4 MGGs). Although they correspond to less than 0.4% of all loci of the reference sequence, they may play very important roles in the survival of the *variant* viruses in each MGG.
  - “Universal” and “MGG-specific HCVGs: There are only 4 HCVGs at loci 241, 3037, 14,408, and 23,403 (see Fig. S3 in B. Supplementary Figures and Tables of the SI and Fig. 4), which are highly conserved in all four MGGs. Thus, they are “universal” HCVGs, and the rest are “MGG-specific” HCVGs. This observation supports some of possible scenarios about the origin of SC2^12,13,and14^, three of them being that (i), since 97% of all MGG I (the first MGG to emerge) members have all four naturally selected HCVGs of MGG I, they were inherited from some of the early *founder(s)* of MGG I, which may have *quickly* acquired the four mutations sequentially or by recombination after a zoonotic transfer event, or (ii) they were acquired while in a nonhuman host, followed by zoonotic transfer to human, or (iii) a combination of (i) and (ii). Our analysis is not sufficient to distinguish them unambiguously.
  - Coemergence of MGG-specific HCVGs: Almost all loci of the HCVGs of each of the remaining three MGGs are not correlated, i.e., they are highly conserved *within* each MGG only, suggesting that the three MGGs have evolved independently, and that their founders emerged at almost the same time (see Observation #8). This suggests that, although each *wave* of infection may appear to occur sequentially, the order of emergence of *the founders* of each wave may not be sequential.
  - Near complete set of MGG-specific HCVGs: Eighty-eight % to 97% of the members of each MGG had the *complete* set of their respective HCVGs (see Table S4 in B. Supplementary Figures and Tables of the SI).
  - Predictability of HCVGs of the next wave: Taken together, these observations suggest that the HCVGs are selected naturally during one wave, and some of the selected HCVGs are inherited in the following waves, or, at least, in the immediate next wave or waves. This implies that one or more sets of the current HCVGs from the four MGGs are *predictable* to become the HCVGs of the new MGG(s) in the next wave, although from which current MGG they will come is not predictable.
    The above genomic characteristics of HCVGs, (a) to (e), suggest a practical approach for designing a new type of vaccine (see “Panvalent” vaccine design for the current and next wave(s) in Implications and Discussion below; see also Fig. 4 and Fig. S3 in B. Supplementary Figures and Tables of the SI).
  - Interestingly, both Fig. 4 and Table 1 show that MGG IV has the largest number of MGG-specific HCVGs (58 HCVGs), mostly due to the presence of many more HCVGs confined to the “spike” gene (S gene) and the N gene (nucleocapsid gene), implying that they may account for MGG IV members’ enhanced infectivity over other variants. Because of MGG IV’s unusual jump in variational changes (most of which are MGG-specific HCVGs) many hypotheses have been proposed on its origin^15–18^.
11. Two Big Bursts in mutational events at the beginning and the end of the pandemic period: Fig. 5 shows a plot combining a box plot and a violin plot of the counts of *mutational events* (compared to the reference sequence) in FESs, not the population counts of all variants, for the four MGGs. There are two jumps (“bursts”) in average mutational events: the first jump at the very beginning of the pandemic when the founder subgroup (containing the reference) gained approximately 18 mutational events, on average, to emerge as all variants of MGG I (minus the reference subgroup); the other jump is at the emergence of the last MGG IV, where the founders of MGG IV also gained 18 mutational events, on average, to emerge from MGG III or 23 mutational events to emerge from MGG II. One of possible suggestion is that the first burst of the variants was for the virus to overcome a new environment, i.e., the human host’s immune system (not yet exposed to SC2), by generating a highly diverse population of viral variants to increase the chance of giving birth to a few highly human-infective variants; the second burst of the variants was to overcome the improved host immune system (which has now gained acquired-immunity by the vaccines and/or natural infections of SC2) by, again, generating a highly diverse unique variant population. The virus succeeded in reaching a pandemic level in the first burst, but not in subsequent bursts so far.
12. HCVGs in a wide range of genomic regions. Table 1 shows that the HCVGs of SC2 are found in the coding as well as non-coding regions of the viral genome, suggesting that they play diverse roles in the survival of the variant SC2s by overcoming the wide spectrum of immune systems of human hosts, such as the antibody-mediated and the cell-mediated immune systems as well as the innate immune system of the host. It is noteworthy, however, that two small regions (the coding region of the S protein, a protein located on the *outer surface* of the virus, and the N protein, the nucleocapsid protein coating the viral RNA *inside* of the virus) have the highest density of the HCVGs. Their locations in the S region suggest that the HCVGs of the region may interfere with the initial interaction between the virus and host cells required for the initiation of viral infection. However, the HCVGs of the N region may have a direct or indirect role in overcoming the host immune system through their involvement in viral assembly, the host cell cycle, interferon-mediated innate immunity and/or other processes^19,20,21^ via non-antibody-mediated mechanism(s).

**At the level of individual variations:**

13. Multiallelic Single Nucleotide Variations (SNVs): When all (including conserved and non-conserved) SNV loci of all MGGs were compared with the reference, an unexpectedly (compared to human SNVs, see below) high proportion of multiallelic SNVs were found in our 44K data. Table S2 in B. Supplementary Figures and Tables of the SI shows that the biallelic SNV loci account for approximately 68% of all SNV loci, the tri-allelic loci approximately 28% and the tetra allelic loci account for approximately 4%. This observation is in contrast to the single nucleotide polymorphisms (SNPs) found in the germline genomes of humans, only other life form for which a massive genomic information is available, where an overwhelming portion of all SNPs turned out to be biallelic^22^.

**Table 1:** Counts of HCVGs in each MGG vs. viral genomic regions.

	5-UTR	ORF1ab	S	ORF3a	E	M	ORF6	ORF7a	betweenORF7a/8	ORF8	betweenORF8/N	N	ORF 10	3-UTR	Total
MGG I	1	2	1												4
MGG II	1	11	9							3	1	7			32
MGG III	2	13	8	1		1		2	1	1	1	5			35
MGG IV	1	13	31	1	1	3	1		1		1	5			58

Although the number of HCVGs in ORF1ab region in each MGG is high, its concentration is low, because the length of the region account for almost 2/3 of the whole virus.

### Implications and discussion: “panvalent” vaccine design against the current and next wave(s)

Some of the demographic characteristics described in Observations section above imply that they can be re-interpreted for the purpose of designing a “panvalent” vaccine against the current as well as the next possible wave of Covid-19:

*“Preview” of the genomic features of the next-wave viruses.* To prevent the next wave of a current endemic or pandemic viral infection, one of the most effective ways would be to vaccinate local or world populations, respectively, against two types of the disease-causing viral strains: (a) one against the current (and most recent past) strains and (b), more importantly, the other against any *future* strains that can bypass the host immune system established for the currently prevailing strains. Experts in the field can come up with good ideas about the first type, but how to design vaccines for the next wave is not usually obvious because of the lack of key information needed to *predict* the genomic features of future viruses. However, with SC2 we have, for the first time, a massive amount of information on the genomic features of the current *and* past SC2 virus variants, their epidemiology, physiology, and others covering *one entire period of the pandemic consisting of multiple waves of the infection*. Such data allow us to perform a “hypothetical preview” experiment, where we position ourselves at a given time point and divide the entire pandemic period into “past/present” and “future”. Then, we ask what highly conserved genomic features in the “past/present” variants are passed to the “future” variants at high frequency.

*Inheritance of HCVGs from MGG I to the remaining 3 MGGs.* As for SC2 shown in Fig. 2, only four MGGs emerged during the pandemic period (see Observation #5 and #6), and, as mentioned in Observation #10, at 4, 32, 35 and 58 loci of MGG I, II, III, and IV, respectively, each locus had an HCVG (approximately 90% or more members of each of the four MGGs had identical and highly-conserved (by, for example, evolutionary selection) variant genotype (see Fig. 4 and Fig. S3 in B. Supplementary Figures and Tables of the SI).

As mentioned in Observation #10b, the 4 HCVGs of MGG I of the first wave are also found in the rest of MGGs of the following waves, suggesting that they are inherited from MGG I. This interpretation suggests that the next new wave(s) may emerge from the member(s) of one or more of the current MGGs carrying their respective HCVGs (see below).

*Predictability of Inheritance of HCVGs from one or more of the four current MGGs to most or all of the next wave viruses*. Based on the pattern of inheritance of the HCVGs mentioned above (see Observation #10(e)), we predict that all or most of the founders of the next-wave viruses will have emerged from one or more of the 4 current MGGs. Therefore, although we cannot predict which members from which MGG(s) will be selected as the founders for the next wave, we can predict that all the HCVGs that will be present in the variants of the next wave will be a subset of the sum of all sets of HCVGs of the four current MGGs.

*HCVGs and the broad spectrum of host immunity*. As mentioned in Observation #12 the HCVGs are found in almost all coding and noncoding regions of the virus, suggesting that they may play an important role in the survival of the variant SC2s, which requires overcoming the *broad spectrum* of the host immune system including innate and adaptive immunity. For example, it is likely that the role of the HCVGs of the S gene (coding for the “spike protein” on the outer surface of the virus) is to help the variants to survive by overcoming, for example, host’s antibody-mediated adaptive immunity, while the role of the HCVGs of the N gene (encoding for the nucleocapsid protein inside the virus) may be to survive by overcoming, for example, the host’s cell-mediated adaptive immunity, innate or/and other types of immunity (^19–21^).

*A new approach for developing vaccines for the next wave:* Since predicting the founder-subpopulation of the new wave from approximately 44,000 unique viral sequences is impossible, we can use a different approach, where we select one viral sequence from each of the four current MGGs, such that the four selected viral sequences contain *all* the HCVGs of the four MGGs, thus, “panvalent” HCVGs. Since one or small number of subpopulations from the current 4 MGGs are expected to emerge as the founder(s) of the next wave all of the next wave viruses would have inherited all the HCVGs of the founding subpopulation. For a more specific example of this approach, see C. “Panvalent” vaccine design” in the SI.

Similar approaches can be applied to design “panvalent” vaccines against infections caused by other viruses, or even by bacterial and other agents if sufficient whole-genome information is available for their variants.

## Materials and methods

For simplicity, brief summaries of the materials and methods used in this study are given below. For the more detailed descriptions see A. Details of Materials and Methods in the SI.

- Curated data of unique variant sequences: We assembled a data set consisting of only “reliable” and unique sequences (see Source data curation in A. Details of Materials and Methods of the SI, and Tables S1 and S2 in B. Supplementary Figures and Tables of the SI) with the earliest sample-collection dates and no duplicates. Our study data set consists of 43,678 unique sequences selected from 406,004 viral sequences.
- Reference sequence: Almost all complete or near-complete SC2 sequences show that the entire coding sequence plus short terminal regions outside of the coding region are highly conserved when compared to the rest (see Fig. S1 in B. Supplementary Figures and Tables of SI). We chose this “extended conserved region (ECR)”, corresponding to the residue number 201–29,749 of the Wuhan-hu-1 strain (Wu-1; Wuhan-Hu-1 (Genbank MN908947) as the reference sequence of the virus for this study (Fig. 5).
- Binary Bit-vector Representation of Sequence: We convert each unique sequence into a binary “bit-vector” representing the identity (0) /difference (1) of the genotype at each position compared to that of the reference sequence, thus, changing sequence data to relational data (see Binary bit-vector representation of genotype variation in A. Details of Materials and Methods of the SI). This vector consists of a large number, 36,735, of elements (0 or 1), representing 25,841 variational events and 10,874 strictly conserved loci, which constitute the complete list of all mutational and conserved sites in the study population. These binary bit-vectors are used to emphasize the genomic variational differences among all variants while reducing the computational load, thus allowing our study of the global grouping of SC2. Such bit-vectors have not been applied in any of the earlier grouping studies of SC2.
- Global grouping: A collection of all such bit-vectors is used for an *unsupervised* clustering by principal component analysis (see Principal Component Analysis (PCA) in A. Details of Materials and Methods of the SI), and the Euclidean “distance matrix” of the bit-vectors is used for a *supervised* clading by Neighbor-joining tree building method (see BIONJ tree in A. Details of Materials and Methods of the SI).

### Supplementary Information

Supplementary Information.

## Data Availability

All genomic data used in this study have been released and are publicly available from NCBI: The NCBI Viral Genomes Resource, http://www.ncbi.nlm.nih.gov/genome/viruses/. No additional new genomic data have been generated from this study.

## Code availability

The codes for variant calling and binary-bit encoding (“one-hot-encoding”) are available at https://github.com/UCB-KIMLAB/sars-cov-2.
