# Supplementary material for: Whole-genome demography of COVID-19 virus during its pandemic period and on “panvalent” vaccine design
Source: Sci Rep. 2024 Aug 1;14:17752. doi: 10.1038/s41598-024-68432-5 (PMC11291670; doi:10.1038/s41598-024-68432-5)
Supplement: Supplementary file 1 — Supplementary Information. [file 41598_2024_68432_MOESM1_ESM.docx]

**Supplementary Information (SI)**

1. **Details of Materials and Methods**

**Source data curation**

We downloaded 406,004 SARS-Cov-2 genomic sequences that are complete and have no unspecified genotype-calls from the NCBI virus hub (The NCBI Viral Genomes Resource, http://www.ncbi.nlm.nih.gov/genome/viruses/) on 2022-02-25, around the time when the last pandemic variants (Omicron) peaked. We excluded 3,023 (less than 1%) genome-size outliers based on the quartile statistics of the whole data:

| **Min.** | **1st Qu.** | **Median** | **Mean** | **3rd Qu.** | **Max.** | **IQR** | **lower**  **boundary** | **upper**  **boundary** |
| --- | --- | --- | --- | --- | --- | --- | --- | --- |
| 2859 | 29763 | 29792 | 29789 | 29817 | 30119 | 54 | 29601 | 29979 |

We also excluded 16,846 sequences (about 4%) that have nucleotide characters of 2-letter or 3-letter IUPAC codes. Parts of 5’- and 3’-UTR regions, 200 and 151 nucleotides respectively, were trimmed off due to high “missingness” (see Fig. S1 in **B. Supplementary Figures and Tables** of **Supplementary Information (SI)**). To identify genomic variations, we aligned each genome against Wuhan-Hu-1 (RefSeq ID: NC_045512.2; Genebank ID of the NCBI Viral Genomes Resource: MN908947.3) and, for each reference locus, identified all the nucleotide(s) different from the reference to make an individual variant-call file containing position, reference genotype, mutation type and variant genotype.

All duplicate sequences are represented by a single sequence with the earliest sample-collection date. Then, any SNP loci where SNP variations that are present only once or twice in the whole population are removed**,** assuming they are weakly supported and possibly experimental errors. We also assumed that each insertion or deletion has occurred by a single event regardless of its length and sequence variation. The final data set contains 43,678 unique sequences (called “the 44K data”).

**Binary bit-vector representation for genotype variation**

We transformed the position-specific genotype-variation data into a binary bit-vector that represents the presence/absence of position-specific mutational-events compared to the reference sequence. This encoding method (Harris, David and Harris, Sarah (2012). Digital design and computer architecture (2nd ed.). San Francisco, Calif.: Morgan Kaufmann. p. 129. ISBN 978-0-12-394424-5.) allows us to efficiently represent and process the variant information into mutational-event information in a computationally manageable format. The following are the details of the workflow.

## Step1. Sequence alignment

## To begin the variant calling process, we aligned each SC2 sequence against a reference sequence using MAFFT (Katoh, Kuma, Toh, Miyata (2005) MAFFT version 5: Improvement in accuracy of multiple sequence alignment. *Nucleic Acids Res*, 33:511-518), a multiple sequence alignment tool implemented in R (Heibl C. (2008). PHYLOCH: R language tree plotting tools and interfaces to diverse phylogenetic software packages. <http://www.christophheibl.de/Rpackages.html>). The following is an example of aligning three sequences: Reference sequence, sample1, and sample2. Each dot represents a consensus nucleotide, and "-" denotes a gap. Each block contains 10 nucleotides. In the alignment output, we can infer that sample1 had 3 mutational events from the reference, named C312A, A319T, AAA insertion after A at position 335. For sample2, there are 3 mutations named A319C, deletion of GCA at position 325, and T329G.

Position 310 320 330

.......... ........... ..........

Reference ..C......A ....GCA...T ....A - - - .....

Sample1 ..A......T ....GCA...T ....AAAA.....

sample2 ..C......C .... - - - ...G ....A - - - .....

Step 2. Variant Calling

Once the alignment was completed, we call variants where the nucleotide sequences are different from the reference sequence by making a variant-specific table for each sample.

Variational-event description.

| **Sample** | **Position** | **Ref** | **Alt** | **Description** |
| --- | --- | --- | --- | --- |
| Sample1 | 312 | C | A | substitution |
| Sample1 | 319 | A | T | substitution |
| Sample1 | 335 | A | AAAA | insertion |
| Sample2 | 319 | A | C | substitution |
| Sample2 | 324 | GCA | --- | deletion |
| Sample2 | 329 | T | G | substitution |

## Step 3. “Binary bit-vector matrix” representation

We converted all variational calls into a “bit-vector matrix (for a simple example, see the table below). Each column's label contains three information: the genotype of the reference at a given sequence position, the sequence position of the reference, and the genotype (“alternative”) of the variant at the sequence position. Since the reference has no genetic variation, all elements of the array for the reference have zeros. For insertions, we consider the last base insertion as the starting position of the insertion, since there are no numbers for insertion sites. Thus, the 3-nucleotide length insertion variant at the 335th position is written as "AAAA".

Binary bit-vector-encoded matrix

| **Variational event** | **C312A** | **A319T** | **A319C** | **GCA324---** | **T329G** | **A335AAAA** |
| --- | --- | --- | --- | --- | --- | --- |
| Ref | 0 | 0 | 0 | 0 | 0 | 0 |
| Sample1 | 1 | 1 | 0 | 0 | 0 | 1 |
| Sample2 | 0 | 0 | 1 | 1 | 1 | 0 |

### **Principal Component Analysis (PCA)**

We conducted PCA analysis (“prcomp” function of R; R Core Team (2022). R: A language and environment for statistical computing. R Foundation for Statistical Computing, Vienna, Austria. https://www.R-project.org/.) on the whole genomic variations encoded by “bit vectors” (Ramnath Vaidyanathan, Yihui Xie, JJ Allaire, Joe Cheng, Carson Sievert and Kenton Russell (2023). htmlwidgets: HTML Widgets for R. R package version 1.6.1. https://CRAN.R-project.org/package=htmlwidgets) as compared to the reference sequence. The bit-encoded rectangular vector matrix consists of 43,678 samples and about 17,000 vector elements, which denote the existence of each mutational event (written in 1 or 0). Colors were assigned by WHO variant names associated with the pangolin lineage: e.g., all the sub-lineages of BA clade and B.1.1.529 are grouped as Omicron. For PCA video, the top 3 principal-component axes were plotted using “plotly” package (C. Sievert. Interactive Web-Based Data Visualization with R, plotly, and shiny. Chapman and Hall/CRC Florida, 2020). Camera angle was updated every second to make the plot spinning using *javascript* and “*htmlwidget*” package. The video was recorded by QuickTime and exported to mp4 video clip by *imovie* program.

### **BIONJ tree**

The BIONJ (a neighbor-Joining phylogenetic tree method; (Gascuel, O. (1997). BIONJ: an improved version of the NJ algorithm based on a simple model of sequence data. Molecular Biology and Evolution, 14(7), 685–695. <http://www.ncbi.nlm.nih.gov/pubmed/9254330>) tree is built using the Euclidean distance matrix as input by summing all pairwise differences between two binary bit-vectors (see **Principal Component Analysis (PCA)** above) for the entire 44K data.

All SARS-CoV2 variants named by World Health Organization (WHO) are indicated on a circular colored-strip. The tree shows that there are four large clades with the overwhelming majority of each clade composed of Omicron (99.9%; MGG IV), Delta (99.4%; MGG III), Alpha (99.9%; MGG II), and the rest of WHO labels plus “Other” (MGG I). The tree is visualized using ITOL (Letunic, I., & Bork, P. (2021). Interactive Tree Of Life (iTOL) v5: an online tool for phylogenetic tree display and annotation. Nucleic Acids Research, 49. <https://doi.org/10.1093/nar/gkab301>). Notice that the founder nodes of the three clades (MGG II, III, and IV) emerged within a short interval on the relative radial-distance scale, which corresponds to the cumulative branch-lengths.

**B. Supplementary Figures and Tables**

**Fig. S1**: “Mutational” rates at 5’ and 3’ terminals; the red arrows indicate the beginning and the ending residue numbers of the “Extended coding region (ECR)” of SC2 sequences used in this study.

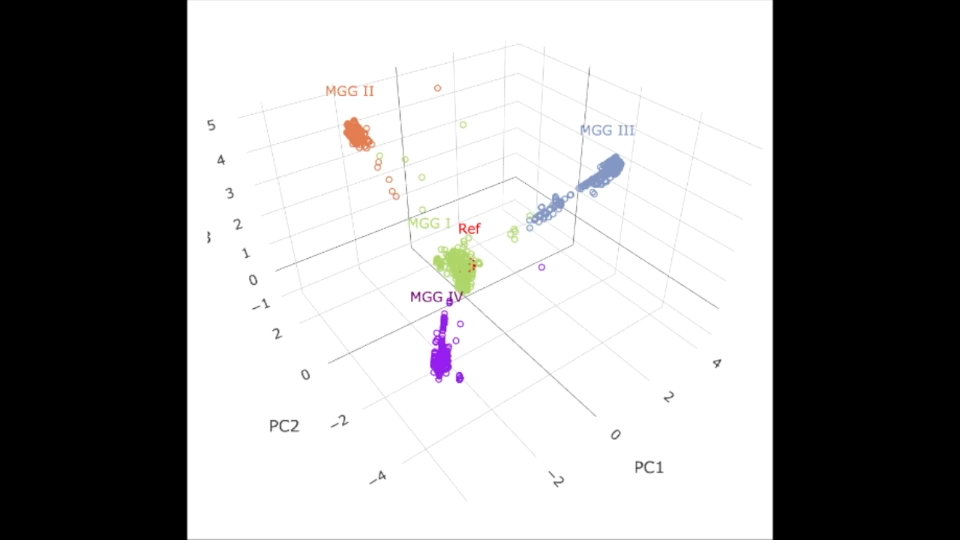


**Fig. S2**: MP4 video (double click to start rotation on the MP4 file). The reference group is shown in red within MGG I.

**Fig. S3**. “Highly conserved (about 90% or higher) variant-genotypes (HCVGs)” in each MGG shown in Fig. 4, where HCVGs are indicated as the vertical lines with small spheres on top.


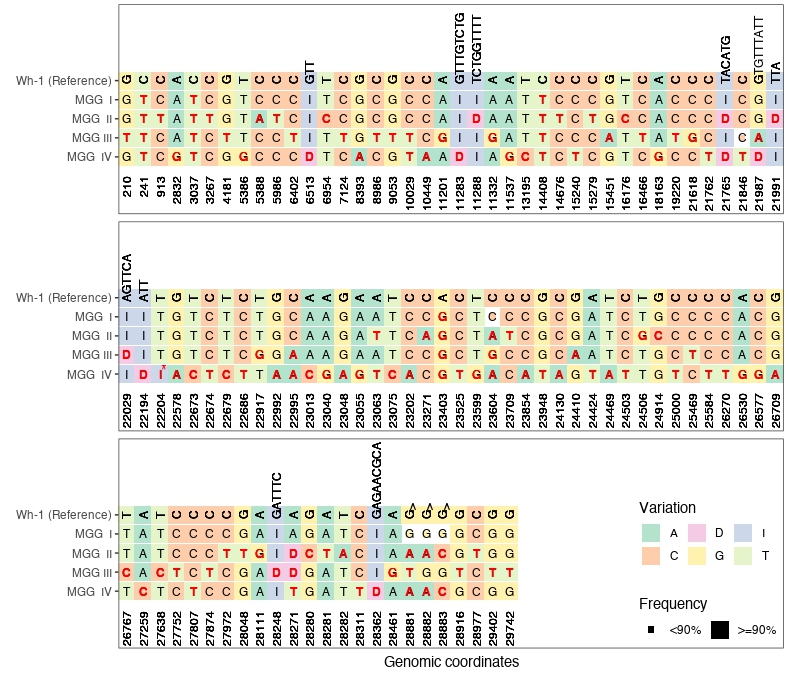


**Table S1**. Filtering of raw data to find the “reliable” unique variant-sequences with earliest sampling dates

|  |
| --- |

Total sequence downloaded from NCBI SC2 dashboard as of 2/25/2022, at the peak of Omicron: 406,004

“Extreme” outliers (based on 3*IQR; see Suppl. Table S1 above): 3,023

Sequences with ambiguous nucleotide calls: 16,846

Remaining data with ECR sequence different from the reference (Wuhan-Hu-1): 386,145

Sequences that have extremely rare genotypes at any sequence positions: 6,042

Collection of the **F**irst (oldest) samples, each among those with the same **E**CR **S**equence (FES): 43,678

Range of the sequence positions of “Extended coding region” in the Wuhan-Hu-1 reference: 201 – 25,749

**Table S2.** Overall feature-counts and diversity of SNVs/Indels in the 44K variant data

|  |  |
| --- | --- |
|  |  |

| \|  \| \| --- \| \|  \| **count** \| \| **Note** \| \| \| Total loci of the ECR of the reference \| 29,549 \| \|  \| \| \| Number of all *unique* variational events, i.e., genotypes of SNVs and Indels \| 25,841 \| \| Dimension of a bit vector used to describe a variant sequence; One loci may have multiple unique variations \| \| \| Number of loci of the reference where variational events occurred \| 18,655 \| \| 63% of the ECR of the reference (Wuhan-Hu-1) \| \| \| Number of loci of the reference where no variational events occurred \| 10,894 \| \| 37% of the ECR of the reference (Wuhan-Hu-1) \| \| \| **Number of SNV sites** \| 18,582 \| \| 342 sites also have INDEL variants \|  \| \| Bi-allelic sites \| 12,560(67.6%) \| \|  \| \| \| Tri-allelic sites \| 5,270(28.4%) \| \|  \| \| \| Tetra-allelic sites \| 752(4.0%) \| \|  \| \| \|  \|  \| \|  \| \| \| **Number of INDEL sites** \| 415 \| \| 4 sites have both insertion and deletion \|  \| \| Type \| # variation \| # n \|  \| \| \| Deletion \| 1 \| 329 \|  \| \| \| Deletion \| 2 \| 39 \|  \| \| \| Deletion \| 3 \| 4 \|  \| \| \| Deletion \| 4 \| 4 \|  \| \| \| Insertion \| 1 \| 39 \|  \| \| \| Insertion \| 2 \| 2 \|  \| \| \| Insertion \| 3 \| 1 \|  \| \| \| Insertion \| 4 \| 1 \|  \| \| |
| --- | --- | --- | --- | --- | --- | --- | --- | --- | --- | --- | --- | --- | --- | --- | --- | --- | --- | --- | --- | --- | --- | --- | --- | --- | --- | --- | --- | --- | --- | --- | --- | --- | --- | --- | --- | --- | --- | --- | --- | --- | --- | --- | --- | --- | --- | --- | --- | --- | --- | --- | --- | --- | --- | --- | --- | --- | --- | --- | --- | --- | --- | --- | --- | --- | --- | --- | --- | --- | --- | --- | --- | --- | --- | --- | --- | --- | --- | --- | --- | --- | --- | --- | --- | --- | --- | --- | --- | --- | --- | --- | --- | --- | --- | --- | --- | --- | --- | --- | --- | --- | --- |
| ­­­**** |

386K data includes all the 44K data (which contain only the variants with unique sequences)

and all the variant populations that have the duplicate sequences of the 44 K data (see Table S1 in

**B. Supplementary Figures and Tables** in **SI).**

| ^1^ The lineages were not labeled in 44K data. | |  |  |  |  |  |  |  |
| --- | --- | --- | --- | --- | --- | --- | --- | --- |
| ^2^ Sample collection Date/Month/Year is not known. |  | | | | | | | |

Table S4

|  | **MGG** | **Total** | ***MaxHCVG*** | ***MinVar*** | ***MaxVar*** |
| --- | --- | --- | --- | --- | --- |
|  | MGG-I | 11986 | 11592 (97%) | MT509667.1 | OL977368.1 |
|  | MGG-II | 8885 | 8509 (96%) | MZ233564.1 | OK234502.1 |
|  | MGG-III | 16324 | 14324 (88%) | OL917159.1 | OL919136.1 |
|  | MGG-IV | 6430 | 5764 (90%) | OM390461.1 | OM800172.1 |

Total: Total count of the members in each MGG within the 44K data; MaxHCVG: Number of the members that have the maximum number of HCVGs in each MGG (4, 32, 35 and 58 HCVGs in MGG I, II, III, and IV, respectively); MinVar: Member ID of the virus that has the minimum number of genomic variations from the reference virus while still having the maximum number of HCVGs in each MGG; MaxVar: Member ID of the virus that has the maximum number of genomic variations from the reference virus while still having the maximum number of HCVGs in each MGG.

1. **“Panvalent” vaccine design**

Some of the demographic observations described in **Observations** section of the main text can be re-interpreted for the purpose of designing a “panvalent” vaccine against the current as well as the next possible wave of Covid-19.

1. **Four MGGs**: During the pandemic period, only four MGGs emerged: MGG I (all WHO-labeled variants minus Alpha, Delta, and Omicron variant groups, and all others not-labeled by WHO); MGG II (Alpha variant groups); MGG III (Delta variant groups); and MGG IV (Omicron variant groups) (see **Observation** #5 and #6 of Main text).
2. **Co-emergence of multiple MGGs:** After the emergence of MGG I, the population centers of the remaining three MGGs appear to show the emergence order may be sequential (MGG I -> II -> III -> IV) on the X-axis in Fig. 1b-1e. However, the same figure also reveals that the first-sampling dates of the *founders* (early samples) of MGG II, III, and IV are almost the same (around Dec. 2020), suggesting that their *founders* emerged from MGG I within a short time range of each other (see **Observation** #8 of Main text). This interpretation implies that the next new wave(s) may emerge from *one or more* of the four MGGs.
3. **Inherited “universal” HCVGs and “MGG-specific” HCVGs:** As mentioned in **Observation** #10 of Main text, there are four “Highly-Conserved Variant-Genotypes (HCVGs)” in MGG I, which are also highly conserved in the remaining three MGGs, thus, they are “universal” HCVGs. Therefore, each of MGG II, III, and IV has a *unique set* of HCVGs (four “universal” HCVGs inherited from MGG I plus 28, 31 and 54 additional “MGG-specific” HCVGs of MGG II, III, and IV, respectively), and one or more sets of the three MGGs are going to be inherited to the future descendant viruses. (This observation also suggests that the three MGGs emerged *independently*, not sequentially, from MGG I, as mentioned in the previous paragraph).
4. **Predictable genomic features of the viruses of next wave:** Taken together, these observations suggest that the next wave will emerge most likely from some sub-populations within about 44,000 unique variants (of MGG I, II, III, and IV combined), where *all* the members of the subpopulation(s) are predicted to carry *all or most* of the HCVGs of respective MGGs.
5. **A new approach for vaccine for the next wave, a specific example of the approach**: Specifically, (a) identify all members of each of MGG I, II, III, and IV; (b) for each MGG, select one sequence that has the maximum number of variant genotypes while keeping the maximum number of the HCVGs; (c) construct a “synthetic” sequence containing all the components necessary to efficiently replicate the variant S and N genes of the selected sequence from each of the four MGGs; (d) use the combination of four such synthetic sequences (in optimized ratio among the four MGGs) as a “panvalent” vaccine. Thus, *such “panvalent” vaccine is expected to induce a broad spectrum of host immunity (innate and adaptive immune systems) against almost all next-wave viral variants, regardless of from which subgroup(s) out of the four MGGs* the founders of the next wave may emerge.
6. Many parameters for effective vaccines, such as stability, efficacy, safety etc., developed, in the case of mRNA-based vaccines, by Pfizer-BioNTech, Moderna, and others, and, in the case of protein-based vaccines by Novavax and others, can also be incorporated in the “synthetic” sequences. Furthermore, to avoid any danger of introducing a new synthetic SC2 virus (a “synthetic live virus”), the candidate vaccines must be limited to a small number of genes not sufficient to self-replicate. For example, the genes coding for the variant S and N proteins are not able to self-replicate, but both have the highest concentrations of HCVGs among all SC2 genes.
7. Similar approaches can be applied to design “panvalent” vaccines against infections caused by other viruses, or even by bacterial and other infectious agents if sufficient whole genomic information is available.
